# Smart Health Evaluation for Lithium‐Ion Battery With Super‐Short‐Segment Charging

**DOI:** 10.1002/advs.202503583

**Published:** 2025-07-27

**Authors:** Qinghua Li, Zhongbao Wei, Hongwen He, Jun Shen, Yang Li, Xiaoguang Yang, Mahinda Vilathgamuwa

**Affiliations:** ^1^ National Engineering Research Centre for Electric Vehicles, School of Mechanical Engineering Beijing Institute of Technology Beijing 100081 China; ^2^ Department of Electrical Engineering Chalmers University of Technology Gothenburg SE‐41296 Sweden; ^3^ School of Electrical Engineering and Robotics Queensland University of Technology Brisbane 4000 Australia

**Keywords:** battery, cycle life, features extraction, machine learning model, SOH estimation

## Abstract

Accurate state of health estimation is crucial for the reliable operation of lithium‐ion batteries in electric vehicles. The charging curve contains valuable features for health evaluation, but real‐world charging often lacks sufficient data due to the users’ early recharging habits. A smart method is proposed for accurate battery health estimation using super‐short charging segments. This method combines a degradation mechanism‐guided Scale‐Invariant Feature Transform for smart health feature identification with machine learning for health evaluation. Validation with 87 batteries with various chemistries, formats, and capacities from 6 manufacturers demonstrates its efficacy. Regardless of battery specifications, health features can be identified automatically from the charging data. The method promises high accuracy (estimation error as low as 1.97%) even with super‐short charging covering 10% state of charge span, where all the existing health feature extraction approaches fail. This method provides new avenues for battery health evaluation in uncertain real‐world electric vehicle applications.

## Graphical abstract

This study proposes a health feature extraction method integrating degradation mechanism‐guided scale‐invariant feature transform with machine learning for lithium‐ion batteries. Validated across 87 commercial cells with diverse chemistries and formats, it achieves 1.97% RMSE in capacity estimation using only 10% charging segments. The approach enables automatic cross‐chemistry feature extraction without requiring complete charging cycles, demonstrating unprecedented adaptability to real‐world partial charging scenarios.

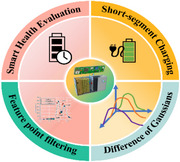

## 1 Introduction

Lithium‐ion batteries are promising in the commercial energy storage sector, especially in the realm of electric vehicles (EVs), due to their high energy density, extended lifespan, and relatively lower costs.^[^ ^1^ ^]^ However, the cycling and calendar aging lead to unpredictable performance degradation of batteries. State of Health (SOH), represented as the ratio of relative remaining capacity to the initial capacity, is utilized as an indicator of battery life status.^[^ ^2^ ^]^ Theoretically, the capacity can be obtained easily by discharging the battery completely. In practical scenarios, however, EV batteries rarely experience complete depletion due to the unpredictable driving and charging habits of EV owners. This aspect further complicates the accurate estimation of SOH.

Various methods have been studied in recent years for SOH estimation. Mechanism models have been developed in the literature to analyze the attributes of battery degradation.^[^ ^3^ ^]^ However, the application of mechanism models for identifying battery health characteristics presents considerable challenges, primarily due to the nonlinear behavior inherent to batteries and the intricate electrochemical reactions.^[^ ^4^ ^]^ To remedy these barriers, data‐driven approaches rely on practical charging data to construct black‐box models for estimating the battery SOH.^[^ ^5^ ^]^ In particular, health indicators are extracted from the measured voltage and current, while machine learning methods like artificial neural network^[^ ^6^ ^]^ and support vector machine^[^ ^7^ ^]^ are employed to map the extracted health indicators towards the battery SOH.^[^ ^8^ ^]^ Sometimes the data‐driven approaches are also combined with lumped battery models for SOH estimation. Illustratively, Yang et al. estimated the impedance parameters with a neural network, and employed the deep convolutional neural networks for online SOH estimation.^[^ ^9^ ^]^

One prominent approach belonging to the data‐driven category is the Incremental Capacity Analysis (ICA), which is employed frequently for extracting and analyzing the battery degradation characteristics from the charge and discharge data.^[^ ^10^ ^]^ Specifically, parameters such as the area under the IC curve and its peak values are considered to be correlated closely with battery aging.^[^ ^11^ ^]^ Previously, a multi‐metric model for SOH estimation was developed based on the health features extracted from IC curves.^[^ ^12^ ^]^ In a similar framework, the joint neural network^[^ ^13^ ^]^ and the probability density method^[^ ^14^ ^]^ were performed based on the IC curve for accurate SOH estimation. Moreover, IC curves were also used to identify the battery degradation mechanisms like the Loss of Active Material (LAM) and Loss of Lithium Inventory (LLI) on the anode and cathode.^[^ ^15^ ^]^

To ensure a reasonable accuracy, the aforementioned data‐driven approaches generally demand lengthy charging data covering the majority of the state of charge (SOC) range. Driven by the EV users’ anxiety to refuel the batteries, however, real‐world charging cannot promise sufficient charging data carrying the required health features.^[^ ^16^ ^]^ For example, many EV users tend to re‐charge the batteries when the SOC is more than 50% to avoid the battery running out of power during traveling. To address this difficulty, health indicators based on partial charging curves, e.g., time interval,^[^ ^17^ ^]^ voltage difference,^[^ ^18^ ^]^ and fuzzy entropy,^[^ ^19^ ^]^ were used in the literature to estimate the SOH with segmental charging data. However, the health feature extraction is generally on a case‐by‐case basis, depending highly on specific analysis for each scenario. Moreover, these methods decline a lot in the estimation accuracy and even lose effect if the charging duration is highly insufficient. In specific, the observable morphological features of IC curve disappear easily when the available charging data is less than 30%.

Another challenge is rooted in the variety of battery materials and specifications, resulting in significant change of the IC curve.^[^ ^20^ ^]^ This means that frequent re‐calibrations are needed for accurate feature extraction once the battery type in use changes. Moreover, the morphological features of IC curve are also highly sensitive to the charging environment. Illustratively, the change of charging current, associated with different charging modes, results in significant morphological discrepancies in the IC curves.^[^ ^21^ ^]^ The uncertainties of IC curve features will cause large errors or even failures of the SOH estimation.^[^ ^22^ ^]^ To address this uncertainty, the extraction of health indicators has to be customized with human intervention, which explicitly violates the practical demand. Hence, an accurate extraction of health indicators adaptive to different batteries and complicated charging conditions is highly desired, yet less explored in the existing body of knowledge.

In this article, we propose a smart degradation mechanism‐guided health feature extraction method that works accurately with the highly‐incomplete charging data (even when 10% of data is available) for various battery types and without any human intervention. The feature extraction is realized by innovatively introducing the Scale‐Invariant Feature Transform (SIFT) algorithm to analyze the IC curves of the battery. The extraction across different space scales is inspired by the changes in the layered structures of graphite anode. The smart feature extraction method is further fused with an ANN model to estimate the battery SOH accurately. Long‐term degradation tests were carried out on small‐capacity 740mAh and 1.1Ah batteries, large‐capacity 40Ah LFP batteries, and ultra‐large‐capacity 280Ah square aluminum shell LFP batteries. Different depths of discharges (DODs) are used to evaluate the impact of incomplete charging on the estimation results.

The proposed method promises two‐fold benefits over the state‐of‐the‐art. First, sufficient health features can be extracted automatically from the short‐segment charging data with this method. Enabled by this, the SOH can be estimated accurately even in an extreme scenario where the charging curve covers only 10% SOC range. By comparison, the existing methods lose effect since the easily‐observed health features disappear under such incomplete charging scenarios. Second, the SOH estimation promises high precision without the need for any human intervention, regardless of the battery chemistries and charging scenarios, which appeals to practical EV applications. This trait prevails over the existing methods, where the feature extraction needs frequent re‐calibration once the battery type or the charging environment changes.

## 2 Results and Discussion

### 2.1 Dataset

Degradation datasets on LCO batteries & LFP batteries with different capacities are constructed in this study. These cells are kept in a temperature‐controlled chamber with different DODs and charging currents. The specifications of batteries are detailed in Table S1 (Supporting Information). The long‐term degradation tests are implemented on all cells with a synopsis of cycling conditions in **Table** **1**. The ambient temperature is maintained at 25 °C, except in cases where it is explicitly stated otherwise. Multiple cells are designated for each cycling condition to observe the cell inconsistency. The testing procedures are as follows:

**Table 1:** Degraded batteries and deteriorating condition for the dataset generation.

Datasets	Cell type	Charge/discharge rate [C]	Depth of discharge [%]	Number of cells
CALCE	LCO battery Type: Prismatic Cells Cutoff Voltage: 2.7V–4.2 V Capacity Rating: 1.1Ah	1/1	100	4
LISHEN	LFP battery Type: LP27148134 Cutoff Voltage: 2.0V–3.65 V Capacity Rating: 40Ah	0.3/0.3 1/1 2/2	100 60 100 60 100 60	3 3 3 3 3 3
CATL	LFP battery Type: CB2W0 Cutoff Voltage: 2.0V–3.65 V Capacity Rating: 280Ah	0.5/0.5	100 60	3 3
EVE	LFP battery Type: LF280K Cutoff Voltage: 2.0V–3.65 V Capacity Rating: 280Ah	0.5/0.5	100 60	3 3
Oxford	Lithium‐ion pouch cells Type: SLPB533459H4 Cutoff Voltage: 2.7V–4.2 V Capacity Rating: 740mAh	1/1	100	8
MST	LFP battery Type: APR18650M1A Cutoff Voltage: 2.0V‐3.65 V Capacity Rating: 1.1Ah	3.7/4 4.8/4 5/4 5.3/4 5.6/4 5.9/4	100	3 8 7 8 16 3

All battery aging cycles were conducted at a controlled temperature (±0.2 °C). The ambient temperature for the Oxford dataset is 40 °C, while that for the MST dataset is 30 °C. The current rate was determined based on the cells' nominal capacity. The depth of discharge of the cells was determined relative to their nominal capacity (100% = 1.1Ah for CALCE battery, 100% = 40Ah and 60% = 24Ah for LISHEN battery, 100% DOD = 280Ah, and 60% DOD = 168Ah for CATL battery & EVE battery).

**Figure 1:**
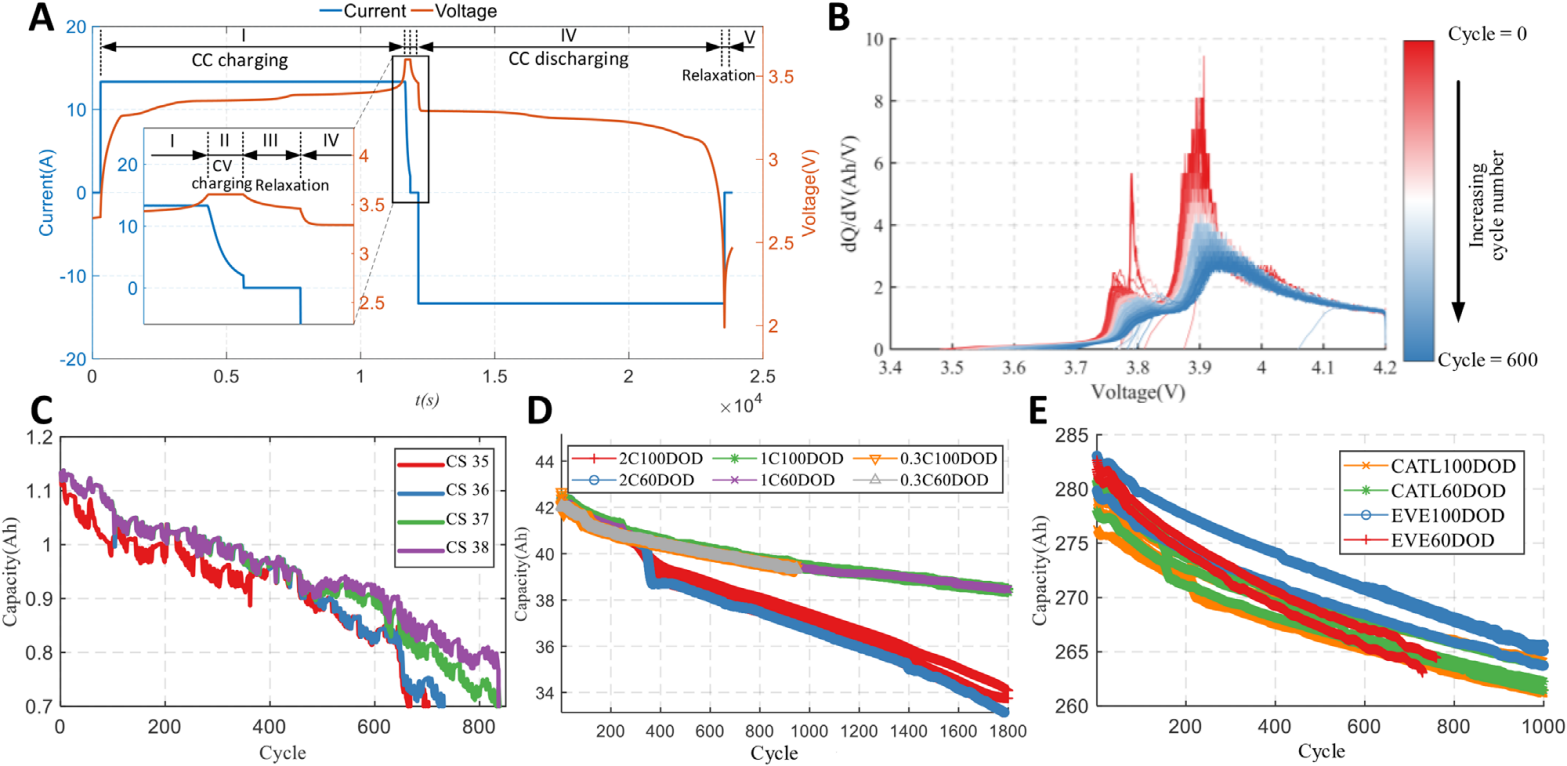
Battery degradation data. Voltage and Current exhibition in the 1th cycle of 0.3C‐100DOD LISHEN battery A). The variation in IC Curve during cycling aging on the CALCE cell B). CALCE battery discharge capacity (until the 70% of nominal capacity) comparison with cycle number of CALCE cell C), LISHEN cell (until the 1800th cycle) D) and CATL & EVE cell (until the 1000th cycle) E). The downward trend (blue curve) in (D) is attributed to the increase of ambient temperature, which is set to 45 °C for a duration of 10 hours to simulate the case of thermal management failure.

Four cells with a nominal capacity of 1.14Ah, labeled as #35, #36, #37, and #38, were selected from the publicly available dataset at the Center of Advanced Life Cycle Engineering (CALCE) at the University of Maryland. These cells were subjected to charging using a CCCV mode, with a charging current of 0.5C, and the upper voltage threshold was 4.2 V. During the CV stage, the terminal current was set to 0.5 mA. Subsequently, these cells were discharged at a rate of 1C until reaching the terminal voltage of 2.7 V. The variation in the cells' capacity with respect to the number of cycles is depicted in **Figure** **1**c. It can be observed that the end‐of‐life point is reached between the 600th and 900th cycles. The entire testing environment was maintained at an ambient temperature of 25 °C. The change in the IC curve during the degradation cycle exhibits a diminishing trend with an increasing number of cycles, as illustrated in Figure 1b.

For LISHEN batteries, cycle‐life tests were conducted until the cell capacity deteriorated to 70% of the nominal capacity. Tests were performed at various rates (2C, 1C, 0.3C) and depths of discharge (100%,60%). For each condition, three cells with a normal capacity of 40Ah were selected. The aging experiments were conducted using the CCCV mode, with charge and discharge rates adjusted according to the respective conditions. Specifically, the complete curve for the initial cycle of the LISHEN battery, operating at a 0.3C rate and with a 100% depth of discharge, is depicted in Figure 1a. This curve comprises five distinct phases: CC charging, CV charging, relaxation, CC discharging, and relaxation. The relaxation time between consecutive cycles is set at 30 minutes. The duration of the CC phase, relative to the other stages, is longer and can be considered as the primary segment. A capacity calibration at a rate of 0.3C is performed per ten cycles to gather capacity variation data at different DODs. The upper voltage threshold was set at 3.65 V during the CC stage, and the terminal current during the CV stage was 0.05C. The ambient temperature was maintained at 25 °C throughout the tests. In order to test the performance of the proposed method under complex conditions, a temperature stress of 45 °C was applied continuously for 10 hours to the 2C100DOD and 60DOD conditions, as depicted in Figure 1d (red curves and blue curves).

EVE's 280Ah square aluminum‐cased LFP batteries and CATL's 280Ah energy storage LFP batteries were employed as test subjects. The CCCV method was applied at an ambient temperature of 25 °C, with the CC stage maintained at 140A and the upper voltage threshold at 3.65 V. The CV stage featured a terminal current of 14A. These cells were then discharged at a rate of 140A until the terminal voltage dropped to 2.0 V. Similarly, every 10 cycles, a capacity calibration test is conducted at a 0.5C rate to obtain information about different DODs. The change in the capacity of ultra‐capacity cells with an increasing number of cycles, as depicted in Figure 1e, demonstrates the distribution of degradation in cycling batteries.

Eight 740 mAh lithium‐ion pouch cells, labeled 1–8, were selected from the publicly available dataset of Oxford University. These cells were charged using the CCCV method with a charging current rate of 1C and a voltage upper limit of 4.2 V. Subsequently, the cells were discharged at a constant current rate of 1C to a cutoff voltage of 2.7 V. The entire testing was conducted at a controlled ambient temperature of 40 °C.

The 1.1 Ah LFP battery dataset from the MIT‐Stanford‐Toyota Research Center (MST) contains extensive aging data under various charging rates. This study focuses on 45 cells charged at constant current rates of 3.7C, 4.8C, 5C, 5.3C, 5.6C, and 5.9C up to the cutoff voltage of 3.65 V, followed by discharge at a constant current rate of 4C to a cutoff voltage of 2.0 V. All tests were conducted at a controlled ambient temperature of 30 °C.

### 2.2 Significance and Challenges of Health Feature Extraction

ICA focuses on morphological features associated with peaks and valleys, such as height, width, and position. These features contain vital information about the battery health, and they are extensively used for SOH estimation of batteries. Specifically, the peaks are labeled as ①, ②, and ③, as depicted in Figure 3b. Each IC peak results from the convolution of electrochemical reactions in the active positive and negative electrode materials.^[^ ^23^ ^]^ The degradation in LIBs stems primarily from various modes, including LLI, LAM, and conductivity loss. Each mode impacts the morphological features of IC curves.

From an electrode perspective, LLI can be rephrased as a slippage or transition of the electrode towards SOC as the capacity gradually diminishes. This slippage is specifically manifested as the translation of peak ① and the shift and degradation of peak ②, which is particularly evident in the LISHEN dataset, as depicted in Figure 3b. In datasets with only partial charge data (Figure 3d), peak 1′s shift and peak 2′s shift and reduction can be observed, where peaks 1 and 2 correspond to peaks 2 and 3 in Figure 3d. The peak shifts caused by LLI also lead to the slippage of peak inflection points, as shown in Figure 3b. The inflection points at 3.3 V exhibit a strong correlation with the number of cycles, while its intensity (peak value) remains relatively stable.

The LAM manifests during the electrode transition process as the battery degrades. The positive electrode transforms into a capacity‐limiting electrode as the battery ages, altering the evolution trend of LAM. Specifically, the last IC peak is diminishing due to the inability to complete the graphite staging. This results in more pronounced peak degradation and an increase in the valley between peaks.^[^ ^24^ ^]^ Observed from the 5–1000th cycle in Figure 3c, the decline in peaks at 3.41 V and 3.44 V is attributed primarily to the LLI, indicating relatively minor degradation during this phase. In contrast, the variation in peak degradation is driven by LAM during the 1000–2600th cycle. During this period, peak ② experiences a drastic degradation, and peak ③ merges with peak ② gradually. The valleys between them also vanish virtually. This transformation reflects the impact of LAM on the electrode transition dynamics.

In spite of its importance, the health feature extraction is extremely challenging. First, different battery chemistries differ remarkably regarding the IC features. As illustrated by the IC curves in Figures 1b and 3b, significant discrepancies are evident in the morphological features of the IC curves. For instance, CALCE cells exhibit two peaks at 3.79 V and 3.9 V, as shown in Figure 1b, while LISHEN cells have three peaks at 3.32 V, 3.41 V and, 3.44 V as depicted in Figure 3b. Furthermore, the 40Ah LISHEN cell (Figure 3b) exhibits a higher IC peak compared to the 1.1Ah CALCE cell (Figure 1b). This arises from the variations in battery capacity, typically resulting in higher peaks for higher capacities. Second, the distinction between Normal Cycle (2C) and Calibration Cycle (0.3C) is evident in Figure 3a, with the peak voltage under low current rates being higher than that under high rates, illustrating a substantial deviation in the IC curve. Third, the DOD also leads to changes in the curve's morphology. This transformation, as reflected in Figure 3b,d, highlights the complexity and variations observed between complete and partial charging segments. Last, the observed peak diminishing and merging phenomenon further increases the complexity of health feature extraction.

### 2.3 Smart Feature Extraction

**Figure 2:**
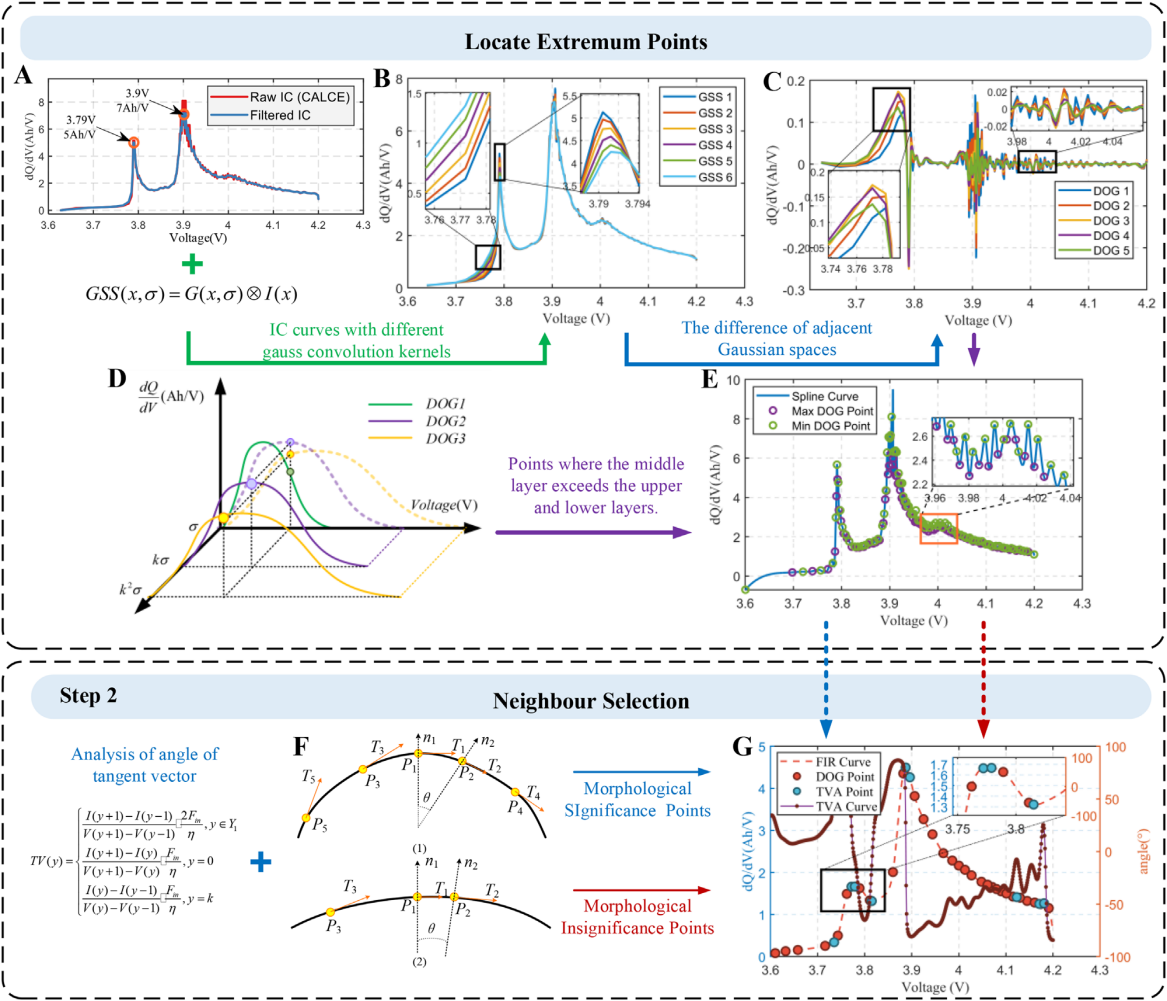
Principles of Adaptive Feature Extraction based on the DoG‐TVA. The Incremental Capacity curves of CALCE cell A).The Gaussian space for Incremental Capacity curves B). and the Difference of Gaussians space on CALCE cell C). The principle D) and results E) of locate the extremum points. The principle F) and results G) of the Neighborhood Selection based on TVA feature.

The aforementioned complexities exacerbate the challenges of aging analysis, necessitating frequent manual adjustments when dealing with new batteries or charging conditions. Moreover, the partial charging condition renders some features unusable, making it impossible to identify the health features even with human intervention. Motivated by this, we propose an automatic feature extraction method capable of adapting to arbitrary operating conditions, as illustrated in **Figure** **2**.

Step 1 focuses on constructing a scale Gaussian space for IC curves (Figure 2b). By differentiating within the scale range, a Gaussian differential space is formed (Figure 2c). Subsequently, extremal points in the scale space related to the voltage range are located, as illustrated in Figure 2d. The purple points on scale *k*σ represent extremal points between adjacent scales σ and *k* ^2^σ.^[^ ^25^ ^]^ Extracting scale features from the Gaussian differential space, as demonstrated in Figure 2e, allows for the identification of curve features such as peak points, valley points, peak start, and end points, which are illustrated in Notes S1, S2 (Supporting Information). However, this method exhibits instability and complexity in extremal points, particularly in the range of [3.96 V, 4.02 V], where noise fluctuations introduce unnecessary scale features. Therefore, a method is needed to re‐filter these points. For this purpose, Step 2 involves the neighborhood selection, measuring the morphological saliency of IC curves by constructing a tangent vector angle curve, as detailed in Note S3 (Supporting Information). It distinguishes between the morphologically salient points (Figure 2f(1)) and non‐salient points (Figure 2f(2)), marked as blue and red points in Figure 2g, respectively. Noise points in flat regions of the curve are marked in red, while the peak points, valley points, and peak start and end points are marked in blue.

With the above two steps, the health features are extracted automatically by analyzing the curve morphology. The computational cost per cycle for the entire process is approximately 0.401 seconds, as detailed in Table S10 (Supporting Information). It is worth noting that the proposed feature extraction method operates automatically without needing to tune any parameters. This eliminates the need for manual intervention or parameter re‐calibration. Unaffected by the battery chemistry and charging condition, the proposed method manifests itself with the capability to identify the morphological features of the IC curve automatically across diverse scenarios.

It is worth noting that the proposed automatic extraction method complies well with the degradation mechanism of LIB. By incorporating diverse scale spaces via Gaussian convolution kernels, the extraction process exhibits notable similarities with the changes in the layered structure of graphite anode. In particular, the features extracted across different space scales resemble distinctions in the graphite structural features, such as the interlayer spacing and lithium‐ion content. Therefore, the automatically‐extracted features virtually reflect the lithium insertion process of LIB, providing deep insights into its internal health state.

### 2.4 Results of Health Feature Extraction

**Figure 3:**
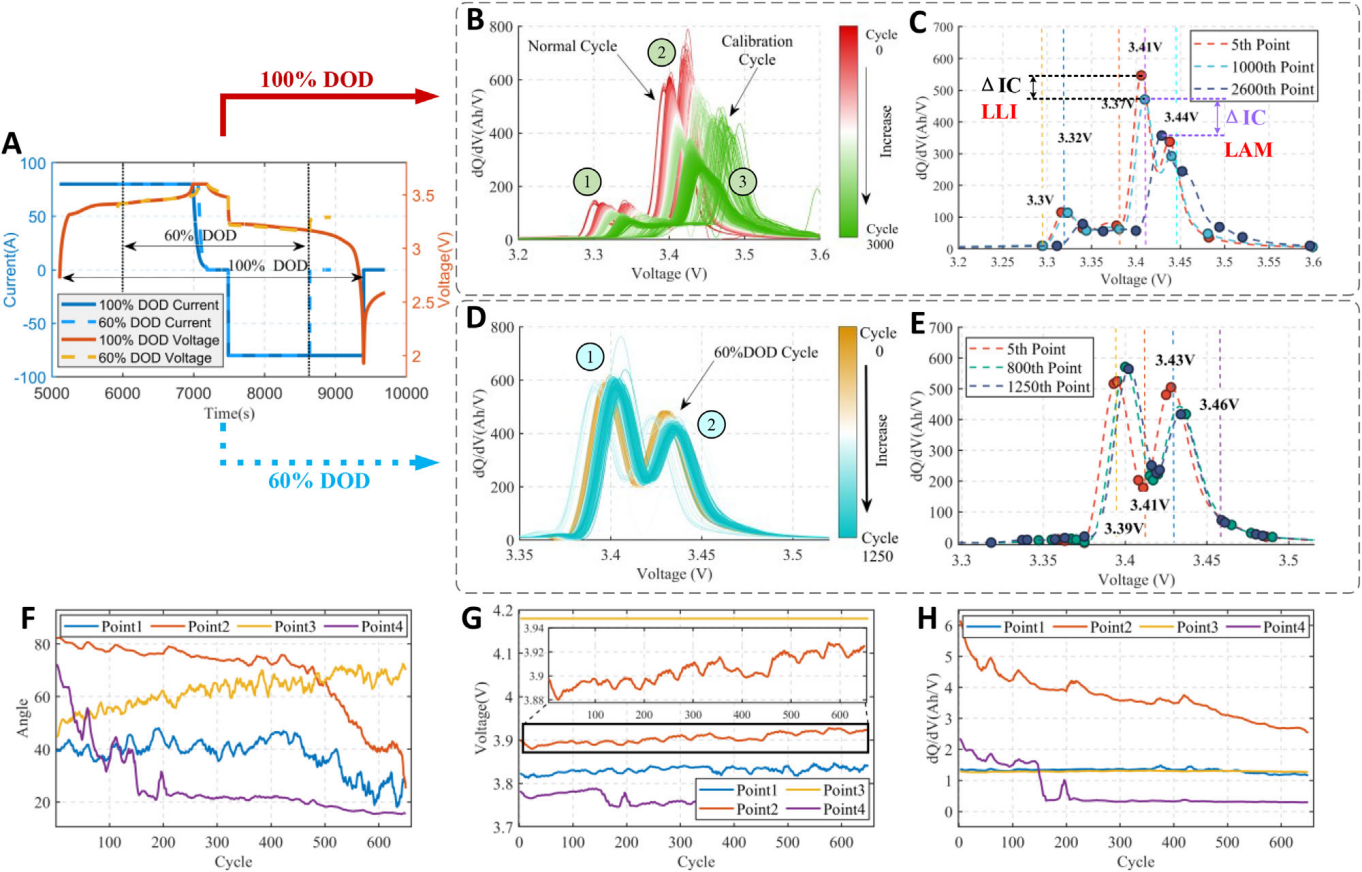
Battery IC curve and DoG‐TVA points. Current and voltage during charging with 100%DOD and 60%DOD A). The change of the IC curve during cycling aging of LISHEN cell: 2C 100%DOD condition B) and 2C 60%DOD condition D). Results of automatic health feature extraction based on DoG‐TVA method for LISHEN cell: 2C 100%DOD condition C) and 2C 60%DOD condition E). The extracted features using DoG‐TVA method in CALCE cells (0.5C 100%DOD condition): Angle F), Voltage G) and Increment Capacity H).

The effectiveness of the proposed feature extraction method can be validated through analysis of **Figure** **3**c. For the complete charging condition, the method demonstrates adeptness in extracting crucial IC features from the 40Ah LISHEN cell (2C100DOD). The features extracted at [3.3 V, 3.32 V, 3.37 V, 3.41 V, 3.44 V] exhibited a notable migration trend as cycles progressed. Furthermore, the method can still extract the health features accurately in adverse scenarios where the battery aging leads to peak merging. This is evidenced by the accurate identification of health features at 3.44 V during the 2600th cycle, as illustrated in Figure 3c. Despite the gradual disappearance of peak ③ and its merge with peak ②, the morphological features can still be delineated appropriately.

As discussed, significant discrepancies exist between the partial charging and complete charging scenarios. Referring to the partial charging condition where some IC peaks disappear, the proposed method still shows the capability of accurate health feature extraction without any manual intervention or parameter recalibration. As illustrated in Figure 3e, [3.39 V, 3.41 V, 3.43 V, 3.46 V] respectively represent the peak, valley, and end point of peaks. Despite the absence of peak ① information at [3.3 V, 3.32 V] in Figure 3c, the proposed method can still identify and capture the morphological features of peak ② and peak ③ accurately, proving its applicability to different charging scenarios and capability of feature extraction with short‐segment charging data.

Correlation analysis is performed to evaluate the significance of extracted features to the battery health. The CALCE dataset is utilized herein, and the changes of extracted Angles, Capacity increments, and Voltage, along with the battery aging, are shown in Figure 3f–h. The specific locations of the feature points are depicted in Figure 1b. Notably, point 2 (the Yellow Line) exhibits a strong correlation with the trajectory of capacity degradation. The Pearson coefficients commonly employed to assess the dependency between the health features and the aging state are summarized in **Table** **2**. The results reveal the significance of the extracted health features, i.e., most of them having a Pearson coefficient over 0.7 and a significant p‐value below 0.05. In our algorithmic framework, the voltage position of the peak is assigned a weighting factor that emphasizes angular features during extraction. Consequently, the weighted peak voltage position does not directly correspond to the highest point of the peak, leading to a lower correlation with battery aging compared to other features. To ensure accurate SOH estimation, the framework integrates angular features, peak characteristics, and morphological features from non‐peak regions. This approach eliminates reliance on a single peak voltage feature.

**Table 2:** Correlation coefficients between DoG‐TVA features and the battery SOH.

Features	#1	#2	#3	#4
Angle (°)	0.7143	0.7708	0.7701	0.7453
IC (Ah/V)	0.5961	0.8531	0.1722	0.6718
Voltage (V)	0.1708	0.1581	0.1560	0.1697

### 2.5 Validation of SOH Estimation

**Figure 4:**
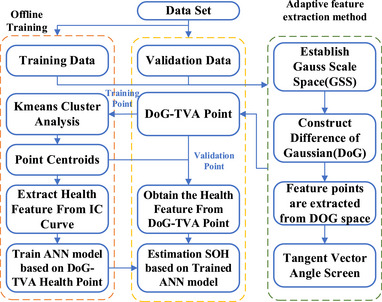
The framework of SOH estimation method. The DOG‐TVA feature points are extracted through the adaptive feature extraction model, and the relationship with SOH is established using a neural network model.

**Figure 5:**
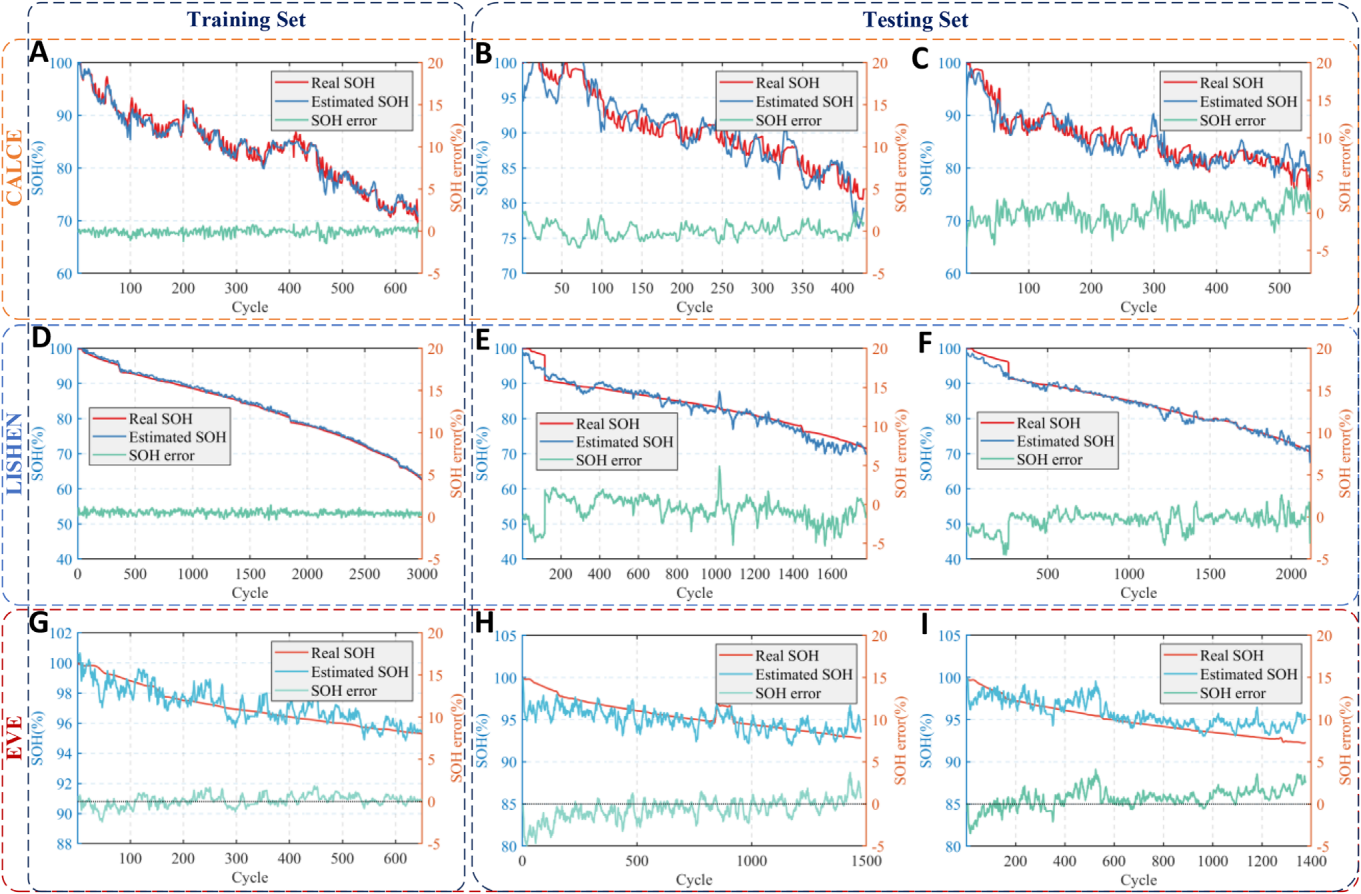
SOH Estimation results for different batteries. For CALCE cells, the SOH estimation on B35 A), B36 B) and B38 C). For LISHEN cells, the SOH estimation on LIB0284 D), LIB0296 E) and LIB 0611 F). For EVE cells, the SOH estimation on LIB1092 G), LIB1859 H) and LIB5726 I).

An ANN model is used herein to estimate the battery SOH with the extracted health features, as detailed in Note S4 (Supporting Information). The SOH estimation method summarized in **Figure** **4** is validated across various scenarios involving multiple batteries and different operating conditions. Using CACLE datasets, the ANN model was trained with data from #CS35 cell and tested with #CS35, #CS36, and #CS38. Results in **Figure** **5**a demonstrate a strong agreement between the estimated SOH and its benchmark. As shown in **Table** **3**, the RMSE of modeling is as low as 0.38%, suggesting the capability of the model to generalize the aging trend accurately. It is worth noting that the batteries from the same batch exhibit certain inconsistencies arising from the manufacturing and aging process. Therefore, the model trained on #CS35 batteries is used to estimate the SOH of #CS36 and #CS38 batteries. As shown in Figure 5b,c, the estimated SOHs resemble their benchmarked trajectories closely. Results in Table 3 suggest that the estimation errors are within 1.5%. This highlights the high robustness of the estimation method to the cell inconsistency.

**Table 3:** RMSEs of SOH estimation with different batteries.

Cell	Current	Train Set	Test Set
CALCE	1C	0.38%	1.35%
LISHEN	2C	0.55%	1.38%
	1C	0.29%	0.93%
	0.3C	1.03%	1.87%
EVE	0.5C	0.97%	1.38%
Oxford	1C	2.31%	1.13%
MST	3.7C	1.03%	1.41%
	4.8C	1.22%	0.54%
	5C	0.65%	1.34%
	5.3C	1.41%	1.78%
	5.6C	1.18%	2.03%
	5.9C	0.96%	0.87%

The estimation method is further validated under different charging currents (2C, 1C, and 0.3C) for LFP batteries (40Ah). The validation processes underwent no manual intervention or parameter re‐calibration, in seeking to validate the automatic feature extraction across various operational conditions.

The results in Figure 5d–f demonstrate an exceptional estimation accuracy, with the RMSE consistently below 2% (Table 3). This illustrates the high generality of the proposed method across multiple operating conditions. For large‐format LFP batteries (280Ah), the estimation results shown in Figure 5g–i and Table 3 indicate an RMSE below 1.5%. For the Oxford dataset, the overall RMSE remains below 2.31%. Similarly, for the MST dataset under minor variations in charging rates, the estimation error is consistently less than 2%. These results demonstrate the proposed method's strong robustness to battery inconsistencies and its adaptability across diverse battery types and operating conditions.

Furthermore, the resilience of the proposed method to environmental interference is tested with measurement noises at 60% and 40% signal‐to‐noise ratios (SNRs). SNRs are employed to partially simulate the noise impact observed in real‐world conditions. It is suggested that the estimation accuracy reduces only slightly even at 40% SNR condition (as shown in Tables S5–S7, Supporting Information). The encouraging results herein validate the high generality of the proposed method to various battery types and charging environments, which is extremely important for practical applications.

### 2.6 SOH Estimation with Incomplete Charging (60% DOD)

In real‐world scenarios, the charging process cannot promise sufficient length of data demanded for extracting the health features, since the drivers generally do not deplete the battery fully before the re‐charging. Therefore, it is paramount to evaluate the proposed method under partial charging scenarios. The performance of the DoG‐TVA method with a DOD of 60% is depicted in Figure S15a–c (Supporting Information). It is shown that the DoG‐TVA features (Figure 3e) differ notably from the case of complete charging (Figure 3c). As depicted in Figure 3e, the charging phases at different DODs exhibit inconsistent trends, resulting in distinct morphological features of IC curves. This dissimilarity primarily manifests as two closely sized peaks at 3.39 V and 3.43 V in terms of morphology. The remarkable deviation of health features in partial and complete charging conditions is attributed to the complexity of degradation mechanisms and the variations in battery phase transition caused by different DODs.

Experimental results on the LISHEN 40Ah cell are illustrated herein, where the cycling is performed with 60% DOD to represent the scenario of partial charging. As illustrated in Figure 3f, the health features are extracted automatically and effectively from the partial charging data without any manual intervention by using the proposed method. Under the 2C, 1C, and 0.3C charging rates, one battery (Cell 1) is selected as the training dataset, while the others (Cell 2 & 3) serve as the testing dataset. The training and testing results are depicted in Figure S15a‐i (Supporting Information), which demonstrate exceptional accuracy. As shown in TABEL 4, the RMSE of SOH estimation remains below 2%, confirming the adaptability of the proposed method to adverse scenarios with incomplete data during the partial charging.

### 2.7 SOH Estimation with Short‐Segment Charging (40% & 10% DOD)

**Figure 6:**
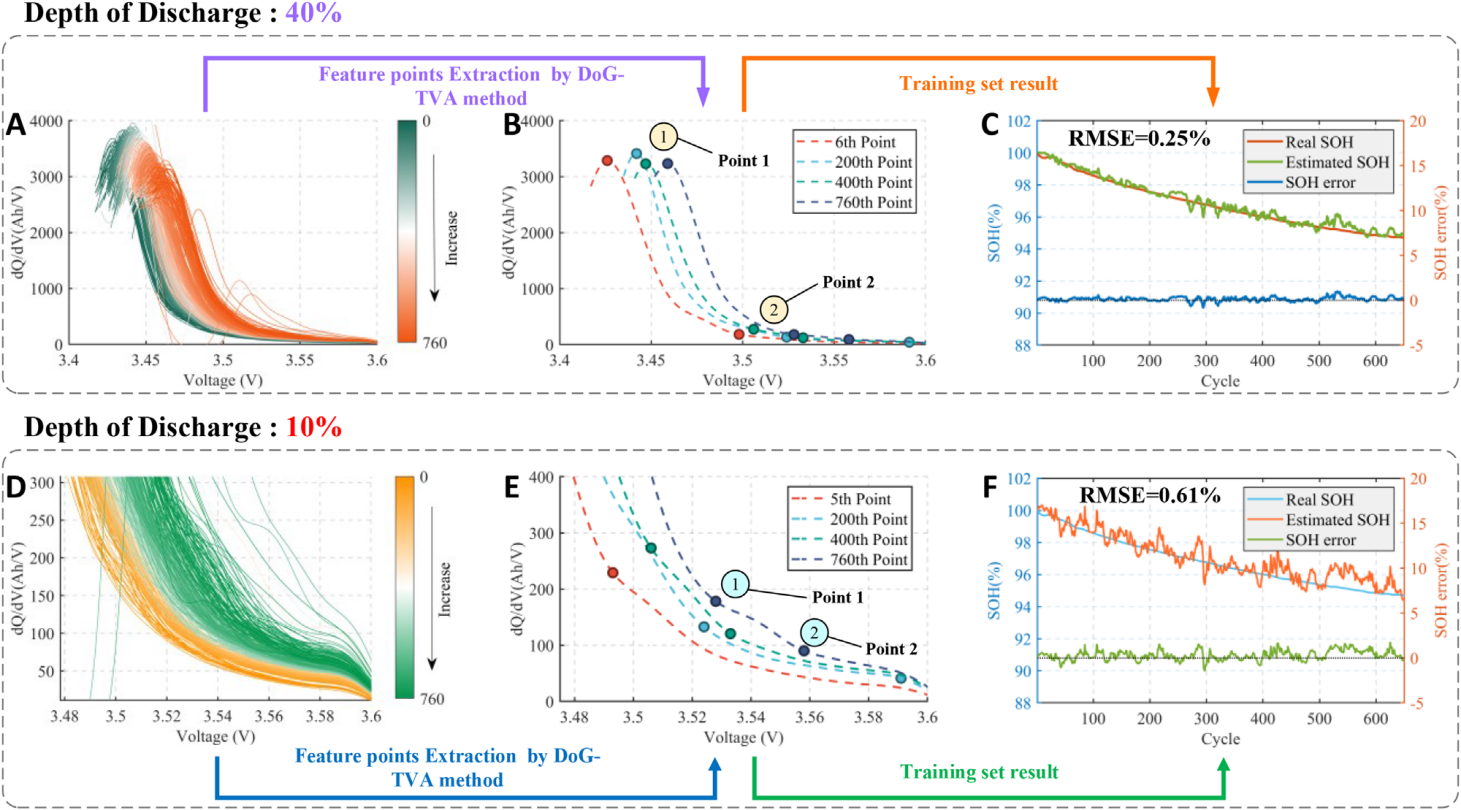
SOH Estimation results with short‐segment charging data. For CATL cells under 40%DOD, the IC curve A), the DoG‐TVA features B) under difference cycle and the SOH estimation on Training set C). And for CATL cells under 10%DOD, the IC curve D), the DoG‐TVA points E) under difference cycle and the SOH estimation on Training set F).

Cycling experiments with 40% & 10% DOD are further investigated to validate the proposed method in heavily‐partial charging scenarios. This means an extremely adverse condition where only short charging segments within 40% & 10% SOC range are available for estimation. The estimated results are depicted in **Figure** **6**a,d.

For the case of 40% DOD, the overall voltage range spans from 3.42 V to 3.6 V. Again, the DoG‐TVA method enables the automatic extraction of key health features, as illustrated in Figure 6b. The extracted health features include Feature Point 1, representing the peak, and Feature Point 2, denoting the end of the peak. Notably, much fewer feature details are extracted in this case compared to the scenario of 60% DOD, resulting in much higher difficulty in SOH estimation. This is witnessed by the deteriorated estimation performance shown in Figure 6c and the corresponding enlarged errors observed in **Table** **4**. In spite of this, the RMSE of 2.33% still suggests a high accuracy concerning such a short‐segment charging condition.

**Table 4:** RMSEs of SOH estimation with short‐segment charging data.

Cell	C‐Rate	Train Set	Test Set
DOD:**60%**	2C	0.15%	1.04%
	1C	0.18%	0.65%
	0.3C	0.11%	1.18%
DOD:**40%**	0.5C	0.25%	2.33%
DOD:**10%**	0.5C	0.61%	1.97%

For the case of 10% DOD, the charging voltage range spans extremely narrow from 3.48 V to 3.6 V. In this severe scenario where only 10% of charging data is available, no peak or valley features can be found on the IC curve, as shown in Figure 6c.

This means that all the commonly‐used morphological clues relevant to battery health in the IC curves are lost completely, resulting in the failure of the broad category of ICA methods. Overcoming this barrier, the proposed method still captures certain features successfully, which are quite different from the IC features in the conventional sense (peak and valley). Moreover, the extracted features, denoted as Point 1 and Point 2 in Figure 6e, are validated with high relevance to the health status of the battery. This can be observed evidently in Figure 6f, where the SOH is estimated with high precision with these new health features. A minor reduction in accuracy and observable fluctuations occur in this case compared to that of 60% DOD charging, as illustrated in Table 4 and Figure S16e (Supporting Information). This is primarily due to the reduced number of traditionally recognized morphological features on the IC curve, which generally decreases with shorter charging segments. In spite of this, the overall estimation error is confined below 5% in such an extreme scenario, justifying the superiority of the proposed method over the state‐of‐the‐art approaches in terms of health feature extraction.

## 3 Conclusion

We propose an accurate SOH estimation method enabled by automatic health feature extraction in this work. With a novel DoG‐TVA algorithm, the proposed method can extract the informative health features smartly, requiring no human intervention or initial parameter setup. By fusing the extracted health features via an ANN model, the battery SOH can be estimated with high precision. The health feature extraction and SOH estimation method adapts to various battery types and complicated charging environments (different C‐rates and DoDs). Impressively, the method can extract sufficient health features for health estimation with short‐segment charging data, even when the charging covers only 10% SOC range. This serves as a major trait since real‐world EV charging is typically incomplete, covering a narrow range of SOC, while the existing approaches fail to find any usable features from short charging segments.

The proposed method effectively addresses challenges posed by extreme conditions such as noise and temperature. Enhancements to the SIFT algorithm and the addition of a Finite Impulse Response filter mitigate noise distortions in the IC curve, resulting in smoother curves with clearer morphological features, as shown in Figure S7 (Supporting Information). Tests with artificially added Gaussian white noise, detailed in Tables S5–S7 (Supporting Information), validate the method's robustness. For thermal variations, controlled experiments were conducted across three temperature regimes: 25 °C for LISHEN, CATL, EVE, and CALCE batteries; 40 °C for Oxford batteries; and 30 °C for MST batteries, demonstrating adaptability to temperature changes. Investigating more complex scenarios, such as dynamic temperature systems and battery pack‐level analyses, remains challenging due to the significantly higher costs, time, and material resources required by the increased system complexity. These challenges are identified as priorities for future research and will be systematically addressed in subsequent studies.

The proposed method has been validated with small (1.1Ah), large (40Ah), and ultra‐large (280Ah) batteries, and has achieved remarkable 1% error rates. Results suggest that the RMSE of SOH estimation is confined within 2.3% with complete charging data for all the batteries from 6 different manufacturers across diverse charging rates. This mitigates the need for frequent human intervention and parameter re‐calibration once the battery type or the charging environment changes. Considering the adverse but practical short‐segment charging scenario (40% or 10% charging segment available), the proposed method can still promise a high estimation accuracy with the RMSE not exceeding 2.33%. This means that the method is applicable to almost all the charging behaviors in real‐world applications.

## 4 Experimental Section

### DoG‐TVA Methods

The automatic feature extraction approach was based on the detection of local feature points in the Difference of Gaussians space. This method eliminates the need for specialized extraction techniques and specific parameter identification.

The establishment of a scale space for the IC curve serves the purpose of simulating the multiscale characteristics present in curve data. Given that the Gaussian convolution kernel was the sole linear kernel capable of effecting scale transformations, the Gaussian convolution kernel was employed for convolving the curve. Its definition was as follows:

(1) $$G\left(x,\sigma \right)=\frac{1}{2\pi \sigma^{2}}e^{-\frac{x^{2}}{2\sigma^{2}}}$$

Where, *G* symbolizes the Gaussian convolution kernel, *x* denotes the battery voltage, *σ* represents the scale which dictates the degree of curve smoothness.

In order to detect stable key points in scale space, a Gaussian Scale Space (GSS) was constructed by convolving a 1D curve with Difference of Gaussian kernels at multiple scales. Consequently, the 1D Scale Space (1D GSS) of IC curve was defined as:

(2) GSS(x,σ)=G(x,σ)⊗I(x)

Here, *I*(*x*) denote the original IC curve, ⊗ represents the convolution operation.

Convolving the IC curve with diverse convolution kernels results in scale‐space curves associated with distinct scenarios, facilitating the representation of the following states:

(3) $$Group_{GSS}\left(x\right)=\left\{GSS(x,\left[\sigma ,k\sigma ,k^{2}\sigma ,\text{…},k^{n-1}\sigma \right])\right\}$$

Here, *Group_GSS_* represents GSS, with *n* denoting the number of group and *k* as the scaling factor, as detailed in Table S2 (Supporting Information). Figure S2 (Supporting Information) analysis reveals significant capacity increases within the IC curve, particularly in the voltage range of 3.76 V to 3.78 V. Notably, the GSS6 curve (light blue) exhibits smoother behavior compared to the GSS1 curve (dark blue), with evident shifts in the peak position of the IC curve between 3.79 V and 3.794 V. These findings underscore the heightened sensitivity of GSS to abrupt curve changes, affirming its suitability for extracting health indicators from IC curves.

Following the construction of Gaussian scale spaces at different scales, *DoG* space for the current scale was generated by computing the differences between adjacent scale spaces. The mathematical expression was as follows:

(4) $$\begin{array}{cc} DoG(x,\sigma ) & =(G(x,k\sigma )-G(x,\sigma ))⊗I(x)\\ & =GSS(x,k\sigma )-GSS(x,\sigma ) \end{array}$$

Here, *DoG*(*x*, σ) represents the *DoG* space for the scaleσ. By computing the differences between adjacent scales within the *Group_GSS_*, the *Space_DoG_* was obtained as follows:

(5) $$Space_{DOG}\left(x\right)=\left\{DOG(x,\left[\sigma ,k\sigma ,\text{…},k^{n-2}\sigma \right])\right\}$$

The number of *Space_DoG_* was one less than the *Group_GSS_*, as illustrated in the Figure S3 (Supporting Information). For instance, in the case of a Gaussian scale space with a scale of 6, the number of Gaussian difference spaces was 5, which was evidently derived.

Figure S3 (Supporting Information) reveals distinct peaks in the range of 3.74 V to 3.78 V, precisely corresponding to the ascent phase in GSS. Furthermore, the pronounced fluctuations at 3.9 V align with the peaks observed in the GSS. Conversely, the oscillations between 3.98 V and 4.04 V on the graph were indicative of noise interference.

Once the *Space_DoG_* was constructed, the next step entails the identification of concealed information points within it. These salient points were constituted by the local extrema in the DOG space. To unearth these distinctive features, a comparison between each data point in the *Space_DoG_* was imperative.

For a given voltage sequence *X* = {*x*_0_,*x*_1_,*x*_2_,…, *x_m_*} in the *DOG*(*x*, σ), there exists ε = 1 such that, for *x* ∈ [1, *m* − 1], all elements in‖*x* − x*‖ < ε satisfy a certain condition denoted by:

(6) $$\begin{array}{c} DOG\left(x^{*},\sigma \right)\geq DOG\left(x,\sigma \right)\\ DOG\left(x^{*},\sigma \right)\leq DOG\left(x,\sigma \right) \end{array}$$

In this context, x*can be regarded as a local extremum point in the *Space_DoG_* at a specific scale. Here, *m* represents the length of the voltage, and ε signifies the range of the local comparison. Apart from assessing extremum points based on voltage, comparing them with points along the scale direction as illustrated in Figure S4 (Supporting Information): In *DOG2*, local extremum point (light purple dot) was compared with its corresponding points in *DOG1*(light green dot) and *DOG3*(light yellow dot). Notably, it becomes apparent that the light purple point exhibits a greater magnitude, identifying it as a salient feature point within the DoG, which can be redescribed as:

(7) $$\begin{array}{c} DOG\left(x^{*},k\sigma \right)\geq DOG\left(x,\left[\sigma ,k\sigma ,k^{2}\sigma \right]\right)\\ DOG\left(x^{*},k\sigma \right)\leq DOG\left(x,\left[\sigma ,k\sigma ,k^{2}\sigma \right]\right) \end{array}$$

The *Space_DoG_* comprises five layers, determined by the six layers of *Group_GSS_*. Notably, it was within the central three layers of *Space_DoG_* that scale and directional comparisons can be conducted. Upon inspecting the local extrema points of the *Space_DoG_* derived from distinct IC curves, as illustrated in the Figure S5 (Supporting Information), that the DoG structure exhibits a discrete nature. This discreteness arises from the dataset construction based on battery charging time. Within a single step, the voltage values were not constant but exhibit fluctuations. This phenomenon was particularly notable at the position of 3.75 V, where the salient points were influenced by the discrete nature of the scale space. As a result, the exact positions of the extrema points may not be precisely determined, often residing in the vicinity of the true extrema. To achieve higher precision in extrema detection, a reconstruction process was necessary.

While the selected DOG feature points effectively mitigate noise interference and address bias issues stemming from discrete sequences, there was still a need for refinement and consolidation of the feature point locations, particularly within dense intervals. Notably, the morphological characteristics of these points were determined by the Tangent Vector Angles (TVA) of the curve, providing an avenue for correction and filtering of imprecise local extrema and dense intervals through TVA analysis.

Establishing corresponding TVA filtering optimization rules to facilitate the morphological feature selection on the curve was imperative. Notably, different batteries exhibit variations in their upper voltage threshold and terminal voltage. Moreover, owing to disparities in battery capacity, incremental capacity also leading to angular deviations. To address this, the concept of a ratio was introduced to express voltage intervals and specific IC values:

(8) $$\eta =\frac{I_{\max}-I_{\min}}{V_{\max}-V_{\min}}$$

Illustratively, *I*_max_ and *I*_min_ denote the maximum and minimum values of the IC curve, while *V*_max_ and *V*_min_ respectively represent the upper voltage threshold and terminal voltage.

In light of this, the Tangent Vector (TV) direction of the IC curve can be defined as follows:

(9) $$TV\left(y\right)=\left\{\begin{array}{c} \frac{I(y+1)-I(y-1)}{V(y+1)-V(y-1)}\cdot \frac{2F_{in}}{\eta },y\in Y_{1}\\ \frac{I(y+1)-I(y)}{V(y+1)-V(y)}\cdot \frac{F_{in}}{\eta },y=0\\ \frac{I(y)-I(y-1)}{V(y)-V(y-1)}\cdot \frac{F_{in}}{\eta },y=k \end{array}\right.$$

Here, *Y*_1_ = 1, 2, …, *k* − 1, *F_in_* represents the input frequency which shown in Table S3 (Supporting Information), *k* denotes the maximum value of the interpolated sequence points. The corresponding angle can be computed using the arctangent formula. Consequently, the tangent vector angle (TVA), can be expressed as:

(10) TVA(y)=arctan(TV(y))

The transformed TVA curve, depicted by the brown line in the Figure S10 (Supporting Information), reveals notable variations at positions where changes in the morphology of the IC curve were more pronounced. Given the substantial degree of variation, this curve can serve as the basis for filtering DOG feature points. The specific selection criteria were shown in Table S4 (Supporting Information).

After TVA curve filtering and optimization, noise‐induced feature points (red points in the Figure S10, Supporting Information) have been removed. The remaining pale blue feature points were better suited for battery SOH analysis and estimation. However, within the 3.75 V to 3.85 V range, multiple points in the same significant feature region have emerged after TVA‐based selection, as seen in the local magnification. These points represent a single peak feature, but their proximity requires additional neighborhood filtering for accurate SOH analysis.

As the cell degradation, noticeable changes in the shape of the IC curve can be observed. As illustrated in Figure S11 (Supporting Information), the initial inflection points at 3.75 V exhibits a rearward shift with increased battery aging, while the peak features at 3.9 V exhibit a downward shift. Notably, the peak features at 3.78 V exhibit both downward and rightward shifts. This trend effectively demonstrates the strong correlation and compatibility between the IC curve and SOH aging, which was poised to play a pivotal role in the SOH estimation process.

Building upon this, K‐means was utilized for clustering the feature point centers, followed by applying the DOG‐TVA feature point selection. Subsequently, the cluster centers were employed for corresponding filtering on all feature points.

The extracted feature points primarily consider their voltage position, peak values, and angular information. Taking the CALCE cell as an example, its feature information, as illustrated in Figure 3f–h, reveals a strong correlation with the cycle count. The correlation between these features and SOH was depicted in Table 2, demonstrating a high level of correlation that validates their substantial potential as parameters for SOH estimation.

## Conflict of Interest

The authors declare no conflict of interest.

## Supporting information



Supporting Information

## Data Availability

The data generated in this study have been deposited in the Zenodo database under accession code: [https://doi.org/10.5281/zenodo.14576042]. And the data processing is performed in python and is available at: [https://github.com/Edward74751/Smart‐Feature‐Identification/].
