# Supplementary material for: Smart Health Evaluation for Lithium‐Ion Battery With Super‐Short‐Segment Charging
Source: Adv Sci (Weinh). 2025 Jul 27;12(36):e03583. doi: 10.1002/advs.202503583 (PMC12463020; doi:10.1002/advs.202503583)
Supplement: Supplementary file 1 — Supporting Information [file ADVS-12-e03583-s001.docx]

Supporting Information

Smart Health Evaluation for Lithium-ion Battery with Super-short-segment Charging

Qinghua Li, Zhongbao Wei*, Hongwen He, Jun Shen, Yang Li, Xiaoguang Yang, Mahinda Vilathgamuwa

**This file includes:**

Supplementary Text: Note 1 to 4

Figs. S1 to S18

Tables S1 to S10

References

Supplementary Text

**Note 1:** **Difference of Gaussians (DoG) Space Based on Incremental Capacity (IC) Curves**

The IC curve of a battery is typically obtained from the ratio of capacity increments to the differential voltage during the Constant Current (CC) stage. It is closely related to the degradation of LIBs and is widely used in the estimation of State of Health (SOH)(*1*). The IC curve is defined as follows:

$I(t)=\frac{dQ(t)}{dV(t)}$ (1)

Here, *I*(*t*) represents the capacity increment, *Q*(*t*) signifies the capacity and *V*(*t*) denotes the voltage at time *t*.

The current utilization and analysis of the IC curve predominantly revolve around peak-related parameters, specifically, the peak height, width, or center position of the curve. These parameters contain critical information regarding the battery's health, necessitating specialized methods or identification parameters for their extraction(*2*). When dealing with new battery types, recalibration and re-extraction of these health-related factors are essential, as illustrated by the orange peaks in the Supplementary Figure S1. The number, positions, and magnitudes of peaks exhibit variations across different battery types. For example, CALCE cells exhibit two peaks at 3.79V and 3.9V, while EVE cells have three peaks at 3.28V, 3.37V, and 3.41V. This inherent complexity adds to the challenge of estimating SOH, demanding frequent parameter adjustments when dealing with new battery types.

To address this issue, this paper introduces an adaptive feature extraction approach based on the detection of local feature points in the Difference of Gaussians (DoG) space(*3*). This method eliminates the need for specialized extraction techniques and specific parameter identification.

The establishment of a scale space for the IC curve serves the purpose of simulating the multiscale characteristics present in curve data. Given that the Gaussian convolution kernel is the sole linear kernel capable of effecting scale transformations, we employ the Gaussian convolution kernel for convolving the curve. Its definition is as follows:

$G(x,\sigma)=\frac{1}{2\pi\sigma^{2}}e^{-\frac{x^{2}}{2\sigma^{2}}}$ (2)

Where, *G* symbolizes the Gaussian convolution kernel, *x* denotes the battery voltage, *σ* represents the scale which dictates the degree of curve smoothness.

In order to detect stable key points in scale space, we construct a Gaussian Scale Space (GSS) by convolving a one-dimensional curve with Difference of Gaussian (DoG) kernels at multiple scales. Consequently, the one-dimensional Scale Space (1D GSS) of IC curve is defined as:

$GSS(x,\sigma)=G(x,\sigma)\otimes I(x)$ (3)

Here, *I*(*x*) denote the original IC curve, represents the convolution operation.

Convolving the IC curve with diverse convolution kernels results in scale-space curves associated with distinct scenarios, facilitating the representation of the following states:

$Group_{GSS}(x)=\left\{GSS(x,[\sigma,k\sigma,k^{2}\sigma,\ldots,k^{n-1}\sigma])\right\}$ (4)

Here, *Group_GSS_* represents GSS, with *n* denoting the number of group and *k* as the scaling factor, as detailed in Supplementary Table S2. Supplementary Figure S2 analysis reveals significant capacity increases within the IC curve, particularly in the voltage range of 3.76V to 3.78V. Notably, the GSS6 curve (light blue) exhibits smoother behavior compared to the GSS1 curve (dark blue), with evident shifts in the peak position of the IC curve between 3.79V and 3.794V. These findings underscore the heightened sensitivity of GSS to abrupt curve changes, affirming its suitability for extracting health indicators from IC curves.

Following the construction of Gaussian scale spaces at different scales, *DoG* space for the current scale is generated by computing the differences between adjacent scale spaces. The mathematical expression is as follows:

$\begin{matrix} DoG(x,\sigma)=(G(x,k\sigma)-G(x,\sigma))\otimes I(x) \\ =GSS(x,k\sigma)-GSS(x,\sigma) \end{matrix}$ (5)

Here, $DoG(x,\sigma)$ represents the *DoG* space for the scale$\sigma$. By computing the differences between adjacent scales within the *Group_GSS_*, the *Space_DoG_* is obtained as follows:

$Space_{DOG}(x)=\left\{DOG(x,[\sigma,k\sigma,\ldots,k^{n-2}\sigma])\right\}$ (6)

The number of *Space_DoG_* is one less than the *Group_GSS_*, as illustrated in the Supplementary Figure S3. For instance, in the case of a Gaussian scale space with a scale of 6, the number of Gaussian difference spaces is 5, which is evidently derived.

Supplementary Figure S3 reveals distinct peaks in the range of 3.74V to 3.78V, precisely corresponding to the ascent phase in GSS. Furthermore, the pronounced fluctuations at 3.9V align with the peaks observed in the GSS. Conversely, the oscillations between 3.98V and 4.04V on the graph are indicative of noise interference.

Once the *Space_DoG_* is constructed, the next step entails the identification of concealed information points within it. These salient points are constituted by the local extrema in the DOG space. To unearth these distinctive features, a comparison between each data point in the *Space_DoG_* is imperative.

For a given voltage sequence $X=\{x_{0},x_{1},x_{2},\ldots,x_{m}\}$ in the $DOG(x,\sigma)$, there exists $\varepsilon=1$ such that, for $x\in[1,m-1]$, all elements in$\left\|x-x^{*}\right\|<\varepsilon$ satisfy a certain condition denoted by:

$\begin{matrix} DOG(x^{*},\sigma)\geq DOG(x,\sigma) \\ DOG(x^{*},\sigma)\leq DOG(x,\sigma) \end{matrix}$ (7)

In this context, $x^{*}$can be regarded as a local extremum point in the *Space_DoG_* at a specific scale. Here, *m* represents the length of the voltage, and $\varepsilon$ signifies the range of the local comparison. Apart from assessing extremum points based on voltage, comparing them with points along the scale direction as illustrated in Supplementary Figure S4: In *DOG2*, local extremum point (light purple dot) is compared with its corresponding points in *DOG1*(light green dot) and *DOG3*(light yellow dot). Notably, it becomes apparent that the light purple point exhibits a greater magnitude, identifying it as a salient feature point within the DoG, which can be redescribed as:

$\begin{matrix} DOG(x^{*},k\sigma)\geq DOG(x,[\sigma,k\sigma,k^{2}\sigma]) \\ DOG(x^{*},k\sigma)\leq DOG(x,[\sigma,k\sigma,k^{2}\sigma]) \end{matrix}$ (8)

The *Space_DoG_* comprises five layers, determined by the six layers of *Group_GSS_*. Notably, it is within the central three layers of *Space_DoG_* that scale and directional comparisons can be conducted. Upon inspecting the local extrema points of the *Space_DoG_* derived from distinct IC curves, as illustrated in the Supplementary Figure S5, that the DoG structure exhibits a discrete nature. This discreteness arises from the dataset construction based on battery charging time. Within a single step, the voltage values are not constant but exhibit fluctuations. This phenomenon is particularly notable at the position of 3.75V, where the salient points are influenced by the discrete nature of the scale space. As a result, the exact positions of the extrema points may not be precisely determined, often residing in the vicinity of the true extrema. To achieve higher precision in extrema detection, a reconstruction process is necessary.

**Note 2: Reconstruct curve and eliminate noise**

Recognizing the challenge posed by inaccuracies in feature points arising from discrete sequences, it becomes imperative to reconstruct IC curves based on voltage sequences, thereby aligning feature points more closely with the actual positions of extrema.

Utilizing 1D piecewise cubic spline interpolation for the voltage sequence $X=\{x_{0},x_{1},x_{2},\ldots,x_{m}\}$, which is divided into *m* intervals. While this method enhances the precision of detected feature points, specifically local extrema, it also introduces an undesirable effect, namely, susceptibility to noise interference. As depicted in the Supplementary Figure S6, an observation of the information between 3.95V and 4V readily reveals a substantial presence of noise within the trend. This noise emanates from the original curve, evidently impacting the extraction of feature points. In this interval, the feature points predominantly correspond to the peaks and valleys of the noise signal, thus posing a potential disruption to the SOH estimation. Consequently, alternative measures need to be employed to mitigate the influence of noise signals.

To address these noises, the spline curve is subjected to filtering by Finite Impulse Response (FIR) filter. Specifically, the signal containing noise is convolved with the Fourier inverse transform values of a low-pass filter. The FIR filter's corresponding parameters are detailed in the Supplementary Table S3.

To simulate real-world noise conditions, Gaussian white noise was superimposed on the original signal. This simulation demonstrates the filter's effectiveness in mitigating such noise interference. It is evident from the power spectral density plot in Supplementary Figure S7 that as the frequency increases, the signal's intensity gradually diminishes. This phenomenon is particularly pronounced beyond the 5Hz threshold. This decrease in intensity signifies the gradual reduction in the strength of the filtered noise components within the signal. This reduction is visually represented in Power Spectral Density (PSD) by the smoothing of the curve, particularly within the voltage sequence range of 300mV to 400mV. In this range, the fluctuations caused by noise interference become notably clearer, as the overall impact of the noise signal is compressed to a minimum.

After applying spline interpolation and FIR filtering, the resulting curve exhibits enhanced smoothness. Furthermore, the selection of local extrema, particularly in the identification of points, has significantly improved in terms of precision and distribution. However, certain discrepancies still persist, as exemplified by the red circular marker in the Supplementary Figure S8. Ideally, it should correspond to the onset of changes in the IC curve's peaks but remain somewhat distanced from this expected position. Additionally, at the 4V location, we encounter an issue of excessive feature points, leading to an overly dense distribution. It is imperative to refine the information associated with these feature points, addressing the problem of excessive density.

**Note 3:** **Neighbourhood Selection Based on Tangent Vector Angle**

While the selected DOG feature points effectively mitigate noise interference and address bias issues stemming from discrete sequences, there is still a need for refinement and consolidation of the feature point locations, particularly within dense intervals. Notably, the morphological characteristics of these points are determined by the Tangent Vector Angles (TVA) of the curve, providing an avenue for correction and filtering of imprecise local extrema and dense intervals through TVA analysis.

Establishing corresponding TVA filtering optimization rules to facilitate the morphological feature selection on the curve is imperative. Notably, different batteries exhibit variations in their upper voltage threshold and terminal voltage. Moreover, owing to disparities in battery capacity, incremental capacity also leading to angular deviations. To address this, we introduce the concept of a ratio to express voltage intervals and specific IC values:

$\eta=\frac{I_{\max}-I_{\min}}{V_{\max}-V_{\min}}$ (9)

Illustratively, *I*_max_ and *I*_min_ denote the maximum and minimum values of the IC curve, while *V*_max_ and *V*_min_ respectively represent the upper voltage threshold and terminal voltage.

In light of this, the Tangent Vector (TV) direction of the IC curve can be defined as follows:

(10)

Here, $Y_{1}=1,2,\ldots,k-1$, *F_in_* represents the input frequency which shown in Supplementary Table S3, *k* denotes the maximum value of the interpolated sequence points. The corresponding angle can be computed using the arctangent formula. Consequently, the tangent vector angle (TVA), can be expressed as:

$TVA(y)=\arctan(TV(y))$ (11)

The transformed TVA curve, depicted by the brown line in the Supplementary Figure S10, reveals notable variations at positions where changes in the morphology of the IC curve are more pronounced. Given the substantial degree of variation, this curve can serve as the basis for filtering DOG feature points. The specific selection criteria are shown in Supplementary Table S4.

After TVA curve filtering and optimization, noise-induced feature points (red points in the Supplementary Figure S10) have been removed. The remaining pale blue feature points are better suited for battery SOH analysis and estimation. However, within the 3.75V to 3.85V range, multiple points in the same significant feature region have emerged after TVA-based selection, as seen in the local magnification. These points represent a single peak feature, but their proximity requires additional neighborhood filtering for accurate SOH analysis.

As the cell degradation, noticeable changes in the shape of the IC curve can be observed. As illustrated in Supplementary Figure S11, the initial inflection points at 3.75V exhibits a rearward shift with increased battery aging, while the peak features at 3.9V exhibit a downward shift. Notably, the peak features at 3.78V exhibit both downward and rightward shifts. This trend effectively demonstrates the strong correlation and compatibility between the IC curve and SOH aging, which is poised to play a pivotal role in the SOH estimation process.

Building upon this, K-means is utilized for clustering the feature point centers, followed by applying the DOG-TVA feature point selection. Subsequently, the cluster centers are employed for corresponding filtering on all feature points.

The computational costs of the improved SIFT algorithm were validated using the MST dataset and analyzed over the final 100 cycles. By recording the actual runtime, the average time per cycle was determined to be 0.401 seconds. Detailed runtime data is provided in Supplementary Table S10. Compared to the duration of a complete charging cycle, the computational time cost is relatively small and within an acceptable range. The algorithm was implemented in Matlab R2023a on a system running Windows 10, equipped with an Intel Xeon Silver 4210R processor and an RTX 3080 Ti GPU.

**Note 4: ANN model of SOH estimation**

The intricate electrochemical reactions and complex mechanisms in LIBs give rise to highly nonlinear behaviors, posing significant challenges for the accurate estimation of SOH using a single health feature. To address this, we employ artificial intelligence neural networks to integrate health features related to angle, voltage, and capacity increment, enabling a comprehensive estimation of LIB's health status. The neural networks utilized in this study for SOH estimation are Backpropagation Neural Networks (BPNN) comprising an input layer with 30 neurons, three hidden layers with 24, 12, and 4 neurons respectively, and an output layer with 1 neuron.

For CALCE batteries, the training and testing results of model are shown in Supplementary Figure S12; for LISHEN batteries, the training and testing results of model are presented in Supplementary Figure S13; for CATL and EVE batteries, the training and testing results of model are depicted in Supplementary Figure S14. For the Oxford dataset battery, the training and testing results of the model are depicted in Supplementary Figure S17, with RMSE errors as shown in Supplementary Table 8. For the MST dataset battery, the training and testing results of the model are depicted in Supplementary Figure S18, with RMSE errors as shown in Supplementary Table 9.

Supplementary Figures

Fig. S1. IC curves for different types of cells (CALCE cells and EVE cells).

Fig. S2. GSS with diverse convolution kernels.

Fig. S3. DoG space with diverse scales.

Fig. S4. Local extremum methods in DoG space.

Fig. S5. Local extremum points in DoG space.

Fig. S6. DoG points in Spline Curve.

Fig. S7. FIR filtered curve and PSD.

Fig. S8. DoG points in FIR filtered Curve.


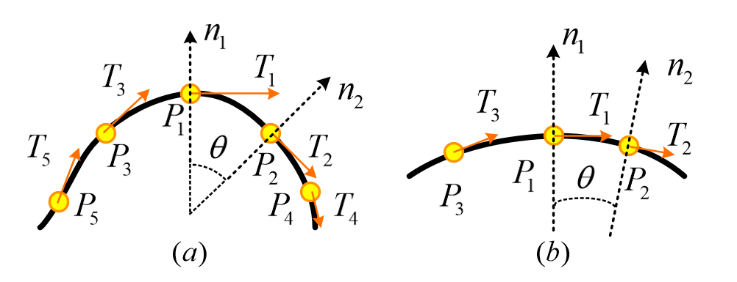


Fig. S9. Flat and protruding as indicated by TVA.

Fig. S10. Changes of feature points after TVA screening

Fig. S11. Changes of DoG-TVA points in different cycles.

Fig. S12. Estimation results on LCO batteries. Train Set: B35 (A) and Test Sets: B36(B), B37 (C), B38(D).

Fig. S13. Estimation results on 40Ah batteries. The results of 2C rate, trained with #LIB0284 (A) and validated using #LIB0296(B) and #LIB0611(C). The results of 1C rate, trained with # LIB0291 (D) and validated using # LIB0813 (E) and #LIB0611(F). And the results of 0.3C rate, trained with # LIB0118 (G) and validated using # LIB0337 (H) and # LIB0448 (I).

Fig. S14. Estimation results on 280Ah batteries. The results of CATL cells, trained with #LIB2763 (A) and validated using #LIB4296 (B) and #LIB4870 (C). The results of EVE cells, trained with #LIB1092 (D) and validated using #LIB1859 (E) and #LIB5726 (F).

Fig. S15. SOH Estimation results under partial charging. For LISHEN cells, the SOH estimation is in the condition of 60% depth of discharge. For 2C rate, the SOH estimation on LIB0248 (A), LIB0034 (B) and LIB 0661 (C). For 1C rate, the SOH estimation on LIB0176 (D), LIB0728 (E) and LIB 0096 (F). And for 0.3C rate, the SOH estimation on LIB0332 (G), LIB2054 (H) and LIB 2061 (I).

Fig. S16. SOH Estimation process with heavy partial charging. For CATL cells, The IC curve in the condition of 100% depth of discharge (A), and the condition of 10% depth of discharge (B). For 10% DOD, the DoG-TVA points (C) and Feature (E) under difference cycle. And the SOH estimation results (D).

Fig. S17. Estimation results on 740mAh batteries. The results of Oxford cells, trained with # 1 (A) and validated using # 2 (B), # 3 (C), # 4 (D), # 5 (E), # 6(F), # 7 (G), # 8 (H).

Fig. S18. Estimation results on1.1Ah batteries. The results of MST cells, For 3.7C rate trained with #LIB 1 (A) and validated using # LIB 2 (B), # LIB 3 (C). For 4.8C rate trained with #LIB 4 (D) and validated using # LIB 5 (E), # LIB 6 (F). For 5C rate trained with #LIB 7 (G) and validated using # LIB 8 (H), # LIB 9 (I). For 5.3C rate trained with #LIB 10 (J) and validated using # LIB 11 (K), # LIB 12 (L). For 5.6C rate trained with #LIB 13 (M) and validated using # LIB 14 (N), # LIB 15 (O). For 5.9C rate trained with #LIB 16 (P) and validated using # LIB 17 (Q), # LIB 18 (R).

Supplementary Tables

Table S1. Specification of CALCE, LISHEN, CATL and EVE batteries.

| **Sample**  **Name** | **CALCE battery** | **LISHEN battery** | **CATL battery** | **EVE battery** |
| --- | --- | --- | --- | --- |
| Battery type | Prismatic LCO Cells | Prismatic LFP Cells | Prismatic LFP Cells | Prismatic LFP Cells |
| Anode material | Graphite | | | |
| Cathode material | LiCoO_2_(LCO) | LiFePO_4_(LFP) | LiFePO_4_(LFP) | LiFePO_4_(LFP) |
| Nominal Capacity | 1100 mAh | 40 Ah | 280 Ah | 280 Ah |
| Nominal Voltage | 3.6 V | 3.2 V | 3.2 V | 3.2 V |
| Cutoff Voltage | 2.7 V - 4.2 V | 2.0 V – 3.65 V | 2.0 V – 3.65 V | 2.0 V – 3.65 V |
| Dimensions | 5.4 x 33.6 x 50.6 mm | 148 x 26.5 x 132.6 mm | 173 x 207 x 71mm | 173 x 207 x 72 mm |
| Battery mass | 21.1 g | 1050g | 5400g | 5420g |
| Reference or Link | https://calce.umd.edu/data | http://baypower.com.cn/en/content/?265.html | https://batteryfinds.com/wp-content/uploads/2021/06/CATL-280Ah-LiFePO4-LFP-Battery-Cell-Product-Specification.pdf | https://www.battery-germany.de/wp-content/uploads/2022/02/LF280K-280Ah-Product-Specification-Version-B.pdf |

Table S1 (continued). Specification of Oxford and MST batteries.

| **Sample Name** | **Oxford battery** | **MST battery** |
| --- | --- | --- |
| Battery type | Lithium-ion Pouch Cells | Prismatic LFP Cells |
| Anode material | Graphite | Graphite |
| Cathode material | - | LiFePO_4_(LFP) |
| Nominal Capacity | 740 mAh | 1.1 Ah |
| Nominal Voltage | 3.6 V | 3.2 V |
| Cutoff Voltage | 2.7 V - 4.2 V | 2.0 V – 3.65 V |
| Dimensions | - | - |
| Battery mass | - | - |
| Reference or Link | https://ora.ox.ac.uk/objects/uuid:03ba4b01-cfed-46d3-9b1a-7d4a7bdf6fac | https://data.matr.io/1/ |

Table S2. Value of parameters in DoG space

| **Parameters** | **Value** |
| --- | --- |
| n | 6 |
| k | 21/3 |

Table S3. Value of parameters in FIR filter

| Parameters | Value |
| --- | --- |
| *F_in_* (Input Frequency) | 1000Hz |
| *F_pass_* (Passband Frequency) | 5Hz |
| Steepness | 0.659 |

Table S4. Specific screening criteria of TVA

| *Initial value: a = 20, b = 20, c = 15* |
| --- |
| ***Step 1*:** For a DOG feature point *P*_1_ after FIR filtering, a comparison is performed with neighboring data points on the filtering curve. The specific comparison interval extends over a range of *a* in both directions, with the feature point *P*_1_ at its center; |
| ***Step 2*:** The TVA value between point P1 and the points within the comparison interval (expressed as normal vectors) signifies the degree of variation within that region. As illustrated in Supplementary Figure S9(a) on the left, a larger angle indicates a more pronounced variation, thereby enhancing the distinctiveness of the feature. Conversely, as shown in Supplementary Figure S9(b), a smaller angle suggests a flatter region. The variation $\gamma$ within this region is represented as; $\gamma=\sum_{i=1}^{\alpha_{\text{num}}} \left\vert\theta_{i}\right\vert/\alpha_{\text{num}}$   Here, $\theta_{i}$ represents the angle between the *i*th point and the reference point, and *a*_num_ signifies the number of adjacent points within the comparison interval |
| ***Step 3*:** When the variation $\gamma$ exceeds 25°, it is considered a distinct feature region and is subsequently retained.  ***Step 4*:** Comparing the points within the region *a* of distinct features, we calculate the degree of variation for each point within the comparison interval. The position with the highest degree of variation is selected as the feature point within the region of distinct features.  ***Step 5*:** Within a radius *c* of *b* neighborhood filtering, the point with the highest variation is kept, and lower variation points are removed. |

Table S5. RMSEs of SOH estimation with different initial signal-noise ratio (LCO batteries)

| white Gaussian noise | B35 | B36 | B37 | B38 |
| --- | --- | --- | --- | --- |
| 100% | 0.38% | 1.38% | 0.86% | 1.32% |
| 60% | 0.39% | 1.43% | 0.85% | 1.56% |
| 40% | 0.52% | 1.55% | 0.96% | 1.55% |

Table S6. RMSEs of SOH estimation with different initial signal-noise ratio (40Ah LFP batteries)

| (2C) white Gaussian noise | LIB0284 | LIB0296 | LIB0611 |
| --- | --- | --- | --- |
| 100% | 0.55% | 1.11% | 1.65% |
| 60% | 0.68% | 1.01% | 1.71% |
| 40% | 0.69% | 1.15% | 1.96% |
| (1C) white Gaussian noise | LIB0291 | LIB0813 | LIB2016 |
| 100% | 0.29% | 0.95% | 0.92% |
| 60% | 0.40% | 1.23% | 1.02% |
| 40% | 0.42% | 1.18% | 1.94% |
| (0.3C) white Gaussian noise | LIB0118 | LIB0337 | LIB0448 |
| 100% | 1.03% | 1.99% | 1.75% |
| 60% | 1.34% | 2.14% | 1.87% |
| 40% | 1.28% | 2.04% | 1.64% |

Table S7. RMSEs of SOH estimation with different initial signal-noise ratio (280Ah LFP batteries)

| (CATL) white Gaussian noise | LIB2763 | LIB4296 | LIB4870 |
| --- | --- | --- | --- |
| 100% | 0.20% | 0.32% | 0.29% |
| 60% | 0.44% | 0.56% | 0.71% |
| 40% | 0.43% | 0.59% | 0.70% |
| (EVE) white Gaussian noise | LIB1092 | LIB1859 | LIB5726 |
| 100% | 0.97% | 1.41% | 1.35% |
| 60% | 0.98% | 1.44% | 1.34% |
| 40% | 1.08% | 1.58% | 1.67% |

Table S8. RMSEs of SOH estimation (Oxford Batteries)

|  | Battery 1 | Battery 2 | Battery 3 |
| --- | --- | --- | --- |
| RMSEs | 2.3143% | 2.1747% | 1.8924% |
|  | Battery 4 | Battery 5 | Battery 6 |
| RMSEs | 1.1394% | 1.1561% | 1.5345% |
|  | Battery 7 | Battery 8 |  |
| RMSEs | 1.2062% | 1.6547% |  |

Table S9. RMSEs of SOH estimation (MST batteries)

| Rate of Current | Train | Test1 | Test2 |
| --- | --- | --- | --- |
| 3.7C | 1.03% | 3.78% | 1.41% |
| 4.8C | 1.22% | 0.54% | 1.24% |
| 5C | 0.65% | 1.74% | 1.34% |
| 5.3C | 1.41% | 1.78% | 2.61% |
| 5.6C | 1.18% | 2.03% | 2.57% |
| 5.9C | 0.96% | 2.00% | 0.87% |

Table S10. Computational Costs of SIFT Evaluated Using the MST Dataset (45 Batteries)

| Number of batteries | Total duration(s) | Single cycle time(s) | Number of batteries | Total duration(s) | Single cycle time(s) |
| --- | --- | --- | --- | --- | --- |
| 1 | 40.5269956 | 0.405269956 | 24 | 39.1645 | 0.391645 |
| 2 | 40.0719949 | 0.400719949 | 25 | 40.90304 | 0.40903 |
| 3 | 39.3656484 | 0.393656484 | 26 | 38.97392 | 0.389739 |
| 4 | 40.0993489 | 0.400993489 | 27 | 40.44688 | 0.404469 |
| 5 | 40.6825205 | 0.406825205 | 28 | 41.30613 | 0.413061 |
| 6 | 38.9334893 | 0.389334893 | 29 | 39.71885 | 0.397188 |
| 7 | 38.5647516 | 0.385647516 | 30 | 40.21915 | 0.402191 |
| 8 | 38.8865363 | 0.388865363 | 31 | 40.53765 | 0.405377 |
| 9 | 39.5412852 | 0.395412852 | 32 | 40.26607 | 0.402661 |
| 10 | 39.4818705 | 0.394818705 | 33 | 40.2644 | 0.402644 |
| 11 | 38.0488926 | 0.380488926 | 34 | 40.44002 | 0.4044 |
| 12 | 38.4336733 | 0.384336733 | 35 | 41.19629 | 0.411963 |
| 13 | 38.4789294 | 0.384789294 | 36 | 41.24201 | 0.41242 |
| 14 | 38.8421953 | 0.388421953 | 37 | 42.04016 | 0.420402 |
| 15 | 39.1921343 | 0.391921343 | 38 | 41.04269 | 0.410427 |
| 16 | 40.4785554 | 0.404785554 | 39 | 40.31557 | 0.403156 |
| 17 | 38.6974518 | 0.386974518 | 40 | 41.69549 | 0.416955 |
| 18 | 39.5627411 | 0.395627411 | 41 | 40.60138 | 0.406014 |
| 19 | 41.5153004 | 0.415153004 | 42 | 40.86178 | 0.408618 |
| 20 | 40.0779103 | 0.400779103 | 43 | 42.34555 | 0.423456 |
| 21 | 38.2273191 | 0.382273191 | 44 | 41.96962 | 0.419696 |
| 22 | 40.6200676 | 0.406200676 | 45 | 41.22544 | 0.412254 |
| 23 | 40.6223045 | 0.406223045 |  |  |  |

Supplementary References

1. X. Li, C. Yuan, Z. Wang, State of health estimation for Li-ion battery via partial incremental capacity analysis based on support vector regression. *Energy* **203**, 117852 (2020).

2. B. Jiang, H. Dai, X. Wei, Incremental capacity analysis based adaptive capacity estimation for lithium-ion battery considering charging condition. *Applied Energy* **269**, 115074 (2020).

3. D. G. Lowe, Distinctive image features from scale-invariant keypoints. *International journal of computer vision* **60**, 91-110 (2004).
